# Effect of transportation and social isolation on facial expressions of healthy horses

**DOI:** 10.1371/journal.pone.0241532

**Published:** 2021-06-04

**Authors:** Johan Lundblad, Maheen Rashid, Marie Rhodin, Pia Haubro Andersen

**Affiliations:** 1 Department of Anatomy, Physiology and Biochemistry, Swedish University of Agricultural Sciences, Uppsala, Sweden; 2 Department of Computer Science, University of California, Davis, California, United States of America; Universita degli Studi di Pisa, ITALY

## Abstract

Horses have the ability to generate a remarkable repertoire of facial expressions, some of which have been linked to the affective component of pain. This study describes the facial expressions in healthy horses free of pain before and during transportation and social isolation, which are putatively stressful but ordinary management procedures. Transportation was performed in 28 horses by subjecting them to short-term road transport in a horse trailer. A subgroup (n = 10) of these horses was also subjected to short-term social isolation. During all procedures, a body-mounted, remote-controlled heart rate monitor provided continuous heart rate measurements. The horses’ heads were video-recorded during the interventions. An exhaustive dataset was generated from the selected video clips of all possible facial action units and action descriptors, time of emergency, duration, and frequency according to the Equine Facial Action Coding System (EquiFACS). Heart rate increased during both interventions (p<0.01), confirming that they caused disruption in sympato-vagal balance. Using the current method for ascribing certain action units (AUs) to specific emotional states in humans and a novel data-driven co-occurrence method, the following facial traits were observed during both interventions: *eye white increase* (p<0.001), *nostril dilator* (p<0.001), *upper eyelid raiser* (p<0.001), *inner brow raiser* (p = 0.042), *tongue show* (p<0.001). Increases in ‘ear flicker’ (p<0.001) and blink frequency (p<0.001) were also seen. These facial actions were used to train a machine-learning classifier to discriminate between the high-arousal interventions and calm horses, which achieved at most 79% accuracy. Most facial features identified correspond well with previous findings on behaviors of stressed horses, for example flared nostrils, repetitive mouth behaviors, increased eye white, tongue show, and ear movements. Several features identified in this study of pain-free horses, such as dilated nostrils, eye white increase, and inner brow raiser, are used as indicators of pain in some face-based pain assessment tools. In order to increase performance parameters in pain assessment tools, the relations between facial expressions of stress and pain should be studied further.

## Introduction

In horses, which are prey animals [1], a multitude of emotional and physical challenges may be present during both ordinary and extraordinary management situations. These situations may include competitions, transportation by road, separation from the herd, social isolation during transportation, introduction to a new environment, and confinement during veterinary diagnostic procedures and treatment. Most of these experiences are putatively stressful and have been shown to induce a physiological stress response [2–4].

Stress is defined as the animal’s non-specific reaction to challenges that require the individual to cope with environmental conditions or psychological challenges [5]. The stress response is a result of the impact from either the environment (external stressors) or the horse itself (internal stressors). An affective component of internal stressors is associated with a stressful experience, generally characterized by a high level of arousal with negative valence [6], thought to be caused by uncontrollability or unpredictability of the animal’s situation [7]. However, internal stressors such as pain may also be relevant. These stress responses are associated with a number of physiological and behavioral changes [8]. In mammals, the response involves activation of two systems, the sympathetic-adrenal medulla axis and the hypothalamic-pituitary-adrenal cortex axis [9]. It may manifest as elevated heart and respiratory rate, blood pressure, and temperature [8]. It may even induce some degree of analgesia [10] or hyperalgesia [11], at least experimentally.

However, many stress-related physiological changes are not specific to stress. Cortisol release shows a diurnal variation [12] and may be affected by pathologies or pain [13]. Heart rate and blood pressure may be elevated in response to purely high-arousal activities, such as exercise [14], or during experience of another affective state, such as pain [13]. This renders physiological markers suitable for measurements of stress in controlled settings, but not in the field, where discrimination between stress and other experiences is important in decision making for both clinical and welfare applications.

Bodily behavioral changes are associated with the fight-flight nature of the horse, while facial behaviors are thought to convey communication to conspecifics [15]. Facial activity can generate a wide array of different observable expressions [16], and has been suggested as a tool for assessment of welfare in mammals [17]. Horses have the ability to generate a remarkable repertoire of facial expressions, which can be described by 17 action units [18]. This is a smaller repertoire than that of humans [16], but larger than that of e.g., chimpanzees or dogs [19,20]. Interestingly, the facial expressions of pain are conserved across mammal species, including humans [21]. It is known that the affective component of pain is expressed by prototypical facial expressions [17]. Recently, it has been shown that horses can display facial changes which are specific to pain [22–24]. However, studies on facial expressions originating from other experiences in horses are very sparse, limiting the use of facial cues for pain assessment in horses since the specificity in relation to other common affective states, e.g., stress [25], is not known. In humans, facial expressions remain a valuable tool for assessing emotional states [16] and furthermore stress induces typical facial expressions [26]. Only a few studies of facial expressions during potentially stressful management situations have been performed in horses, most focusing on features around the eye [27] or blinking frequency [28,29]. In order to address other facial features during these interventions, a tool called the Equine Facial Action Coding System (EquiFACS) [18] can be used. EquiFACS records facial expressions by observing onset and offset of anatomically based action units (AUs) and action descriptors (ADs) over time. The method does not infer anything about the meaning of facial movements observed, leaving less space for subjective judgment. The resulting dataset contains spatio-temporal data on the occurrence of different AUs, time of onset, offset, and duration, and their temporal overlap with other active AUs.

These datasets tend to be large, even with relatively small sample sizes, and thus they are difficult to classify without manual interference. To determine AUs that are typical for pain in humans, methods based on frequencies of AUs have been proposed [30]. However, statistical methods for analyzing FACS data on animals are not yet well-developed. To address this problem, use of data-driven machine learning principles has been applied for the analysis of facial expressions [31]. Such methods have been proven feasible when analyzing other large and variable datasets in the biological sciences, for example behavioral studies [32]. In a recent study, EquiFACS data were used for determination of facial expressions of pain [33]. Using a machine learning method utilizing the temporal overlap of AUs in observation windows of different lengths, co-occurring facial expressions which discriminated between painful and non-painful horses were determined in a very small dataset in that study (N = 6) [33]. The results of this method largely agreed with those of the frequency-based method, but it was also able to identify less frequent, but distinct, AUs of relevance for pain [33]. To our knowledge, exploration of facial expressions originating from affective states using this or other methods has yet to be explored in horses, and it is not clear whether facial expressions of pain in horses can be affected by other cognitive states.

The aim of this study was therefore to describe facial expressions during two common horse management events which putatively induce a physiological stress response in healthy individuals. Based on clinical and ethological descriptions during similar events, we expected to identify facial action patterns, with the most prominent being changes in repetitive mouth behaviors, flared nostrils, flattened ears [34], and the ADs yawning and tongue show [35]. We also expected an increased number of AUs in response to visual or auditory inputs, displayed as increased frequencies of ear movement and eye blinks [28,29,36]. To our knowledge, a complete set of EquiFACS facial expressions in these situations has not been described previously.

We further hypothesized that the frequency methods applied in human research can identify important AUs and ADs in horses, but that methods using temporal distribution (co-occurrence of facial expressions) are important, especially when frequency and duration of distinct facial traits are low or environmental input is high. Finally, we explored whether facial expressions during the interventions can be classified using a Linear Support Vector Machine, to support the construct validity of the facial expressions selected by the two methods.

## Materials and methods

### Ethical statement

This study was approved by the Ethics Committee for Animal Experiments in Uppsala, Sweden (Approval no. 5.8.18-10767/2019). Owner consent for the use of privately owned horses was obtained before experimentation.

### Study design

For this study, consisting of one observational part and one experimental part, two standard horse management practices were used: short-term transportation and short-term isolation. These interventions are generally considered to be linked to psychologically induced stress. Video footage was recorded during the events, and during the horses’ normal living conditions before or after the intervention. A body-mounted, remote-controlled heart rate monitor provided continuous heart rate measurements in all three situations.

### Study groups

A total of 28 horses were used in the study. A heterogeneous study group, consisting of 18 privately owned horses (PRI), was included. They comprised 10 geldings, seven mares and one stallion, of the breeds Thoroughbreds (n = 5), mixed-breed ponies (n = 4), Standardbred trotters (n = 3), and Swedish warmblood/riding breeds (n = 6), with body weight ranging between approximately 400 and 600 kg. The median age of horses in this group was 10 years (range 3–24 years). They were considered healthy by their caretakers, had not been subjected to veterinary treatment for the previous two months, and had not been treated with analgesics during that period. The horses were managed at home, by the horse owner, in the routines to which they were accustomed. Most of these horses had previously been introduced to transportation. All were kept in stables except for the thoroughbreds, which were kept in a free-range system. Three of the PRI horses were kept at the university but were treated as though they were privately owned.

A more homogeneous study group was included from the university herd (UNI), consisting of nine Standardbred trotters (seven mares and two geldings) and one warmblood mare. They were considered healthy at routine examinations during the previous four months, were of median age 12 years (range 8–19 years) and had roughly similar body weight. They were kept in an authorized research facility at the Swedish University of Agricultural Sciences. These horses were fed hay four times a day, and oats once a day according to a nutritional plan that supported normal condition. All horses were allowed out on pasture for 6 hours a day and otherwise kept in individual 3 m x 4 m boxes. The horses were transported once a year to summer pasture, but other than that not regularly accustomed to transportation. During the experimental part, horses were moved to other boxes in the same facility and acclimatized for at least 16 hours. Horses were moved together in pairs, stabled besides each other, and kept in their regular stable herd (together for at least the previous six months). Each pair of horses had the same feeding and housing routine and had the same caretakers in all stables.

Horses in the two study groups, PRI (N = 18) and UNI (N = 10), all underwent the transportation intervention. The PRI horses were studied in their own stable and were transported in their own trailer. The UNI horses were transported in a standard horse trailer, which was novel for the horses, for 20 minutes. All UNI horses showed reluctance to enter the trailer and some loading procedures took up to 30 minutes before the horses entered the transport. All horses from UNI (N = 10) were used to create a subgroup, which in addition was subjected to social isolation on a subsequent occasion. Social isolation was performed by taking out the herd mate, leaving the horse alone in the stable for at least 15 and at most 30 minutes. The horses were kept in the same box as during the control intervention, making the environmental factors the same.

### Video-recording

Video-recordings of the horses were made during the two interventions and during baseline without the presence of an observer. During the transport intervention, video-recordings were made in the box and inside the horse trailer, using GoPro Hero 3+ Silver Edition and GoPro Hero 7 Black cameras (Gopro Inc., San Mateo, California, USA). Resolution was set to 1080p at 30 fps and videos were exported to mp4-format. The cameras were mounted depending on the layout of the box, so that the entire horse and its box could be seen in the footage. If the stable had no regular box, the horses were filmed in their grooming spot. In the trailer, the halter of the horse was tied to a front bar in a standard manner, and the camera was mounted in line with the horse’s head height and angled approximately 45–60 degrees from the horse’s medial plane. The cameras recorded for 10 to 20 minutes during transportation, and for at least 30 minutes during baseline.

During the experimental social isolation intervention and during the baseline for the UNI subgroup, the horses were filmed in their own boxes. These video-recordings were made using two wall-mounted standard surveillance cameras with night vision (WDR EXIR Turret Network Camera, HIKVISION, Hangzhou, China). Extra light was provided with nine standard fluorescent lights mounted in the ceiling, programmed to provide light during daytime hours. The cameras were mounted in each corner in the front of the box so only the horse and its box could be seen in the footage, in order to ensure blinding. Resolution was set to maximum and images were exported to mp4-format. The cameras recorded all baseline sessions for a minimum of 30 minutes and social isolation sessions for a minimum of 15 minutes.

### Heart rate monitoring

A remotely controlled heart rate monitor (Polar Wearlink, Polar Electro OY, Kempele, Finland), made for equine use, was used to obtain continuous heart rate measurements without the interference of an observer. The Wearlink device was fastened using a girth, which was soaked in water before attachment. Heart rate measurements started well before the interventions and the horses were allowed to adjust to the transmitter for at least 10 minutes before filming began [37]. The heart rate monitor was time synchronized with the videos, using a gesture in the video when the transmitter was started or using the time-stamped files produced by the cameras and heart rate transmitter. Files containing R-R intervals were exported through Polar ProTrainer Equine Edition (Polar Electro OY, Kempele, Finland). Anomalies in the heart rate measurements were removed using the program’s own algorithm with the medium filter and minimum protection zone of six beats per minute. Heart rate measurements were extracted as a mean during five minutes, with onset two minutes and 15 seconds before the 30 second annotation clip and offset two minutes and 15 seconds after the clip ended. A Wilcoxon signed rank test was used to calculate significance in the PRI group. In the UNI group, a Wilcoxon signed rank test was used to test for the specific rise in heart rate between the baseline and the respective intervention. In the latter, the p-values were corrected according to the Bonferroni-Holm method.

### Video processing and annotation

The identity of the video-recordings of the transportation group could only be blinded for horse, and not for intervention, since the location in the trailer and its movements could not be hidden. Selection of clips was made by manual inspection and 30-second clips of suitable footage were cut from the videos. If the face was visible and scorable for more than 30 seconds, a random number generator was used for video selection.

The identity of the video-recordings from the experimental social isolation intervention was blinded in relation to horse and intervention before annotation. Selection of videos for the social isolation group was performed using an automated horse face detection software [38], where sequences were selected if the head position of the horse was visible and suited for annotation. Thirty-second sequences of video with a side- or front-view confidence of at least 60% were selected. If several selections were available, a random number generator was used to select one clip. The selected clips were manually inspected to ensure that the software had successfully identified a face. If not, a new clip was randomly selected.

All films were annotated in a blinded manner by two EquiFACS-certified state-approved veterinarians with a minimum of 70% correct annotations compared with expert raters. All transportation and baseline films were also annotated by one of the authors (JL), who is also certified in EquiFACS. Annotation was performed using a template consisting of all codes in EquiFACS, including supplemental codes and the visibility code VC74 (code for unscorable), but without head movements (AD51-AD55). Annotation was performed with the open-source program ELAN [39]. The annotators coded the onset and offset of the facial AUs, allowing calculation of frequency and duration, i.e., how frequently an AU or AD occurred and how long it remained active. The annotators set the onset of the AU to when the muscle started contraction and the offset to when it was fully back to neutral again. Inter-rater agreement between the coders was calculated using the Wexler ratio as described by Ekman et al. [16], using all 30-second clips. Inter-rater agreement was found to be on average 0.75 (coder 1–2: 0.76; coder 2–3: 0.76; coder 1–3: 0.71), indicating good agreement between raters.

### Selection of EquiFACS codes

Since inter-rater agreement was good, one set of annotations was randomly selected and used for each video. For each selected AU or AD, frequency and duration were observed. It was anecdotally noted that the frequency of ear-related ADs had a high presence in the dataset. In order to determine whether these movements were due to the ear moving back and forth or the ears focusing on a certain point, a facial movement index (FMI) was created. To describe *ears forward* (EAD101) and *ear rotator* (EAD104) occurring together within a one-second interval the term “ear flicker” was used. The FMI was created prior to any hypothesis testing where the EquiFACS codes were selected. It is important to note that this is not an AD, but an index describing a series of specific facial movements (ADs) that occur in succession to constitute the “ear flicker”.

EquiFACS codes and the “ear flicker” were analyzed using the method described by Kunz et al. [30], here called the Human FACS Investigation (HFI) method. Action units that accounted for more than 5% of total AU occurrences in stress videos were selected. From this subset, AUs detected at higher frequency in the intervention videos than in control videos were selected as the final set of intervention AUs. While the HFI AU selection method ensures that selected codes are frequent and distinct, they may have only a slightly stronger correlation with the experienced state and can exclude less frequent, but highly discriminative, AUs. Therefore, the relative temporal distribution of AUs was also considered. In order to do this, the method of Rashid et al. [33], here referred to as the Co-occurrence method, was used to calculate the co-occurrence of AUs. This method selected EquiFACS codes that occurred together with other EquiFACS codes more frequently in stress than in no-stress states. Since onset and offset of EquiFACS codes were recorded in ELAN, codes which appeared simultaneously or in close relation to each other could be further studied. EquiFACS codes that occurred within a predetermined period (observation window size, OWS) were recorded as co-occurring. Action units that exhibited the largest difference in co-occurrence patterns between intervention and control were selected. The method uses directed graphs to record and calculate differences in co-occurrence patterns. Furthermore, a paired t-test for mean values was used to test significance, with p<0.05 considered significant.

For both the HFI and Co-occurrence methods, occurrences of *ears forward* (EAD101) and *ear rotator* (EAD104), which were included in the “ear flicker” category, were not double-counted for EAD101 and EAD104 separately. As a result, occurrence counts of EAD101 and EAD104 did not occur within a one-second interval of one another.

### Classification of facial expressions during the interventions

The EquiFACS codes selected by the HFI and Co-occurrence methods were used to train a machine learning classifier, Linear Support Vector Machine (LSVM), for intervention versus control classification. Twenty-five control videos and 35 intervention videos (10 from social isolation, 25 from transportation) were used. The frequency and duration features in the clips were used to represent each video sequence, in order to train the LSVM for the classification. This was done without the “ear flicker” adjustment, in order to have pure data. Using five-fold cross-validation, the optimum regularization parameter *C* and balanced class weights were selected. The Python Scikit-Learn library [40] and the Leave-One-Out (LOO) protocol were used to train and test the models, meaning that the features of all videos except one were used to train an LSVM, which then used the same features on the remaining video to determine whether it showed a stressful intervention. The LSVM predictions were collated across the entire dataset, and precision and recall were calculated. Precision was reported as the proportion of true positives in the total number of predictions and recall as the proportion of true positives which could be identified by the model. Overall accuracy, the number of correct predictions in the number of total predictions, was also calculated. The performance of the LSVM models indicated how well the selected EquiFACS codes captured the facial expressions during transportation and social isolation, thus acted as a type of construct validity to classify the interventions.

## Results

### Heart rate during interventions

**Fig 1:**
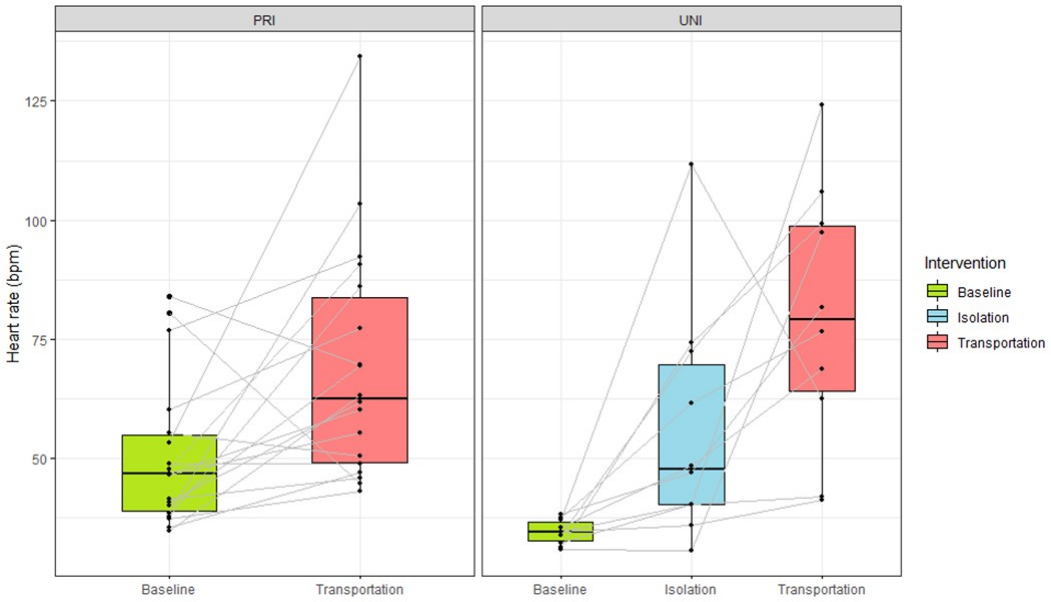
Heart rate during interventions. Boxplots showing the heart rate of (left) privately owned horses (PRI) and (right) university horses (UNI) during baseline, social isolation, and transportation.

The means of the five-minute heart rate periods during interventions are shown in Fig 1. For the PRI group, heart rate increased from a pooled mean of 54 bpm (SD 25.5) during baseline to 77 bpm (SD 32.3) during transportation (p = 0.008). For the UNI group, heart rate increased from 35 bpm (SD 4.4) to 65 bpm (SD 30.5) during social isolation (p = 0.008) and to 88 bpm (SD 31.5) during transportation (p = 0.004), meaning that all interventions caused a rise in heart rate compared with the control situation.

### Selected annotations

A full set consisting of 7900 annotations was created from the films. The horses displayed higher frequencies of facial movements during the interventions than during baseline, with the horses having an average of 38 annotations during social isolation and 57 annotations during transportation, compared with 26 annotations during baseline.

#### HFI method

Action units which comprised at least 5% of AUs recorded during the interventions, and the percentage difference between interventions and control, are presented in Table 1. The results for the transportation and social isolation interventions are presented both separately and combined, to display differences and similarities between the groups. Generally, similar codes were selected in both groups with the exception of *Upper lid raiser* (AU5) and *Half blink* (AU47), which were not selected during social isolation, and *Ear rotator* (EAD104), which was selected only during social isolation. Blink AUs (AU145 and AU47) and *inner brow raiser* (AU101) had the most similar rate of occurrence between intervention and control, while *eye white increase* (AD1), *nostril dilator* (AD38) exhibited the largest difference in frequency between intervention and control recordings. However, an AU not selected during social isolation, *upper lid raising* (AU5), exhibited the largest difference in frequency between transportation and control recordings. The movement index “ear flicker” was also more frequent and more pronounced in transportation than in social isolation.

**Table 1:** Facial expressions during the interventions as defined by the HFI method.

	*Eye white increase* (AD1)	*Nostril dilator* (AD38)	*Inner brow raiser* (AU101)	*Blink* (AU145)	*Half blink* (AU47)	*Upper lid raiser* (AU5)	“Ear flicker”	*Ear rotator* (EAD104)
**Transportation**								
**Percentage of AUs during intervention / control**	8.2% / 4.8%	13.1% / 8.4%	5.3% / 8.1%	12.7% / 19.6%	7.7% / 11.2%	8.0% / 5.7%	18.9 / 17.7%	Not selected
**Difference in frequency**	113.7%	106.2%	31.4%	30.1%	35.8%	98.6%	76.2%	Not selected
**Social isolation**								
**Percentage of AUs during intervention / control**	7.8% / 3.9%	15.0% / 7.0%	15.0% / 12.1%	18.1% / 19.8%	Not selected	Not selected	16.6% / 20.2%	5.3% / 6.2%
**Difference in frequency**	85.7%	90.9%	43.0%	12.8%	Not selected	Not selected	1.9%	6.1%
**Combined**								
**Percentage of AUs during intervention / control**	8.2% / 4.8%	13.4% / 8.4%	7.2% / 8.1%	13.8% / 19.6%	8.0% / 11.2%	7.0% / 5.7%	18.4% /17.7%	Not selected
**Difference in frequency**	106.8%	101.8%	52.1%	29.3%	30.5%	80.7%	66.4%	Not selected

Action units (AUs) and action descriptors (ADs) selected using the Human FACS Investigation (HFI) method to represent stressful interventions in horses in the transportation and social isolation groups and together as a combined group.

When combining both groups, all AUs that comprised at least 5% of stress AU occurrences were also more frequent during intervention videos than control videos. Other than the exceptions mentioned earlier, the chosen AUs for social isolation were identical to those selected for transportation stress, but the percentage difference between control and intervention frequency counts was noticeably larger for *inner brow raiser* (AU101).

#### Co-occurrence method

Action units and ADs selected using the Co-occurrence method are presented in Table 2. Of the selected codes, *nostril dilator* (AD38), *tongue show* (AD19), *mouth open* (AU25), *upper lid raiser* (AU5), *eye white show* (AD1), and “ear flicker” showed significance in all OWS. *Inner brow raiser* (AU101) was selected by the HFI method and significant (up to a 5-second OWS) using this method.

**Table 2:** Facial expressions during the interventions (combined) as defined by the Co-occurrence method.

OWS	*Inner brow raiser* (AU101)	*Lips part* (AU25)	*Tongue show* (AD19)	*Nostril Dilator* (AD38)	“Ear flicker”	*Blink* (AU145)	*Eye white increase* (AD1)	*Nostril lift* (AUH13)	*Upper lid raiser* (AU5)	*Half blink* (AU47)	*Ears forward* (EAD101)	*Ear rotator* (EAD104)
**2**	✓	✓	✓	✓	✓	✓	✓	✓	✓			
	(p = 0.042)	(p<0.001)	(p<0.001)	(p<0.001)	(p<0.001)	(p<0.001)	(p<0.001)	(p = 0.064)	(p<0.001)			
**5**	✓	✓	✓	✓	✓	✓	✓	✓	✓	✓		
	(p = 0.024)	(p<0.001)	(p<0.001)	(p<0.001)	(p<0.001)	(p<0.001)	(p<0.001)	(p = 1.000)	(p<0.001)	(p = 0.107)		
**10**	✓	✓	✓	✓	✓	✓	✓	✓	✓	✓		
	(p = 0.051)	(p<0.001)	(p<0.001)	(p<0.001)	(p<0.001)	(p = 0.013)	(p<0.001)	(p = 0.450)	(p<0.001)	(p = 0.185)		
**15**	✓	✓	✓	✓	✓	✓	✓	✓	✓	✓	✓	
	(p = 0.052)	(p<0.001)	(p<0.001)	(p<0.001)	(p<0.001)	(p = 0.037)	(p<0.001)	(p = 0.576)	(p = 0.001)	(p = 0.373)	(p = 0.173)	
**20**	✓	✓	✓	✓	✓	✓	✓	✓	✓	✓	✓	
	(p = 0.090)	(p = 0.001)	(p = 0.001)	(p<0.001)	(p<0.001)	(p = 0.053)	(p<0.001)	(p = 0.383)	(p = 0.003)	(p = 0.238)	(p = 0.217)	
**30**	✓	✓	✓	✓	✓	✓	✓	✓	✓	✓	✓	✓
	(p = 0.179)	(p = 0.018)	(p = 0.017)	(p<0.001)	(p = 0.001)	(p = 0.079)	(p<0.001)	(p = 0.450)	(p = 0.018)	(p = 0.210)	(p = 0.252)	(p = 0.641)

Action units (AUs) and action descriptors (ADs) selected using the Co-occurrence method to represent the interventions in horses using different observation window sizes (OWS).

### Frequency and duration patterns

**Fig 2:**
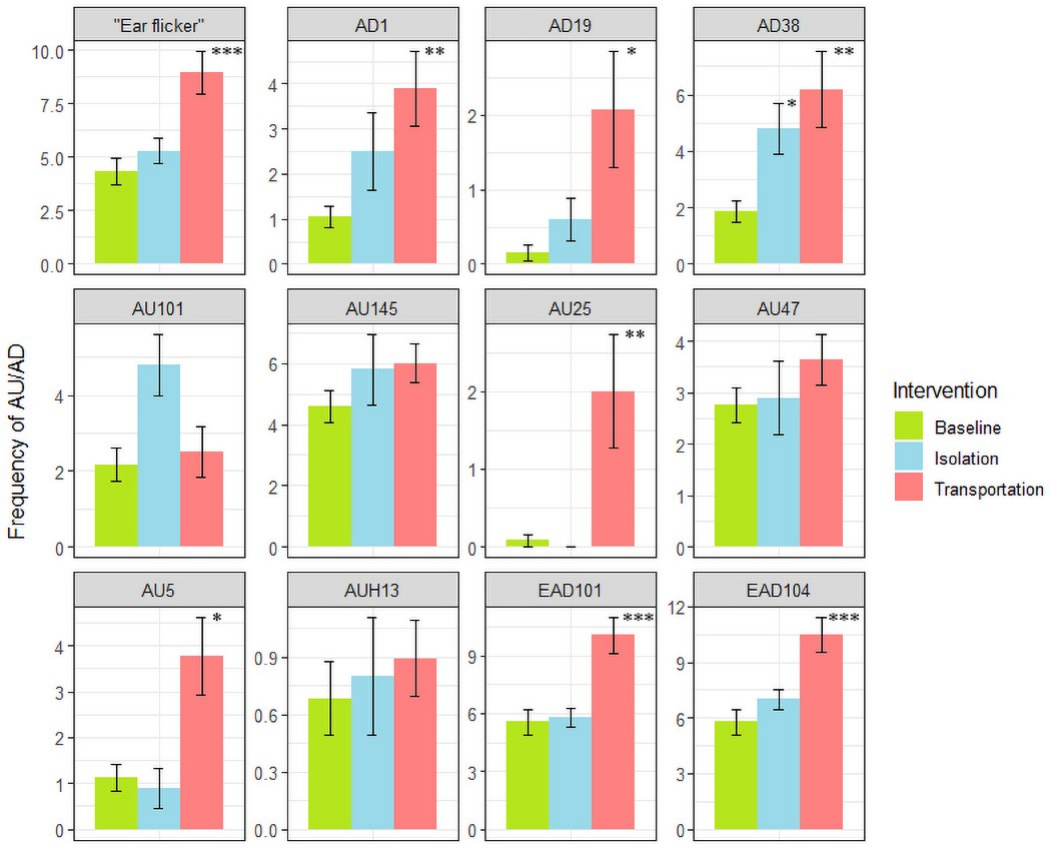
Frequency of EquiFACS codes. Changes in action unit (AU) and action descriptor (AD) frequency patterns between interventions and control. Asterisk marks significant difference from control (*p<0.05, **p<0.01; ***p<0.001).

**Fig 3:**
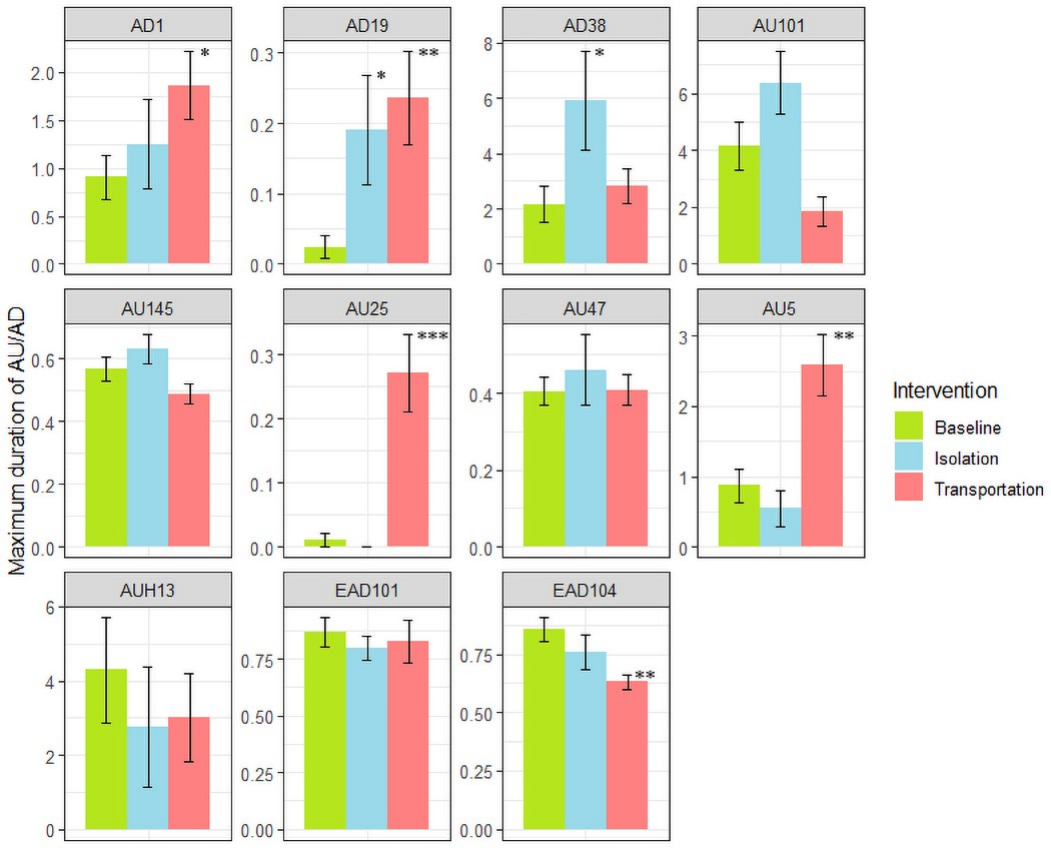
Maximum duration of EquiFACS codes. The interventions affected the duration (s) of activity for an action unit (AU). Asterisk marks significant difference from control (*p<0.05, **p<0.01; ***p<0.001).

In order to study each facial expression in detail, average frequency and maximum duration patterns for the above selected ADs are further presented in Figs 2 and 3, respectively. Action unit frequency increased for eight of the selected codes, mainly during transportation. With just 10 horses in the group, AU frequency was rarely significant for isolation stress. Only *Nostril dilator* (AD38) increased in frequency during social isolation. *Inner brow raiser* (AU101), despite its high frequency in the social isolation intervention, was not statistically significant. All AUs selected by the HFI method had p<0.01 for at least one representation and intervention. Additionally, *tongue show* (AD19) and *lips part* (AU25), which were only selected by the Co-occurrence method, showed p<0.01 across all groups and representations, for either frequency or maximum duration, when tested separately.

### Leave-One-Out classification

The selected AUs in Tables 1 and 2 were used to train the LSVM for stress or no-stress classification, in order to check the validity of the selected AUs. The results of the LOO classification are presented in Table 3, which shows both the precision (proportion of positive predictions that were correct), and recall (proportion of positive instances that were correctly predicted). Accuracy (proportion of test instances correctly predicted, both positive and negative), is also shown. The best classification was obtained using both frequency and maximum duration, reaching an impressive 95% recall rate for the AUs selected by the HFI method and 78% precision rate for the AUs selected by the Co-occurrence method. Interestingly, the model was able to classify between the interventions and control almost as accurately even without selection of important AUs, although the precision and recall were better when focusing on a few AUs.

**Table 3:** Results of Leave-One-Out classification for action units with and without pre-selection.

	Frequency	Max duration	Both
**HFI method**			
**Precision**	75.56%	66.67%	75.00%
**Recall**	89.47%	68.42%	94.74%
**Accuracy**	77.27%	62.12%	78.79%
**Co-occurrence method**			
**Precision**	73.68%	66.67%	73.91%
**Recall**	73.68%	68.42%	89.47%
**Accuracy**	69.70%	62.12%	75.76%
**Without selection of AUs**			
**Precision**	71.79%	62.86%	76.74%
**Recall**	73.68%	57.89%	86.84%
**Accuracy**	68.18%	56.06%	77.27%

## Discussion

This study investigated whether common putatively stressful management procedures can induce facial expressions in horses, and whether these expressions can be recorded and identified using an objective facial coding system that exhaustively codes all facial activity, and not only predetermined actions. Transportation and social isolation were selected as interventions, since both are well described in the literature and practice, increasing the relevance of the study for horse management [41,42]. However, the literature mainly concentrates on the physiological or clinical component of the stress response or welfare issues during transportation and isolation [42], while the emotional characteristics are less well described. According to the dimensional approach, the dimensions of arousal and valence should be considered, but this is a particular difficulty in non-verbal species [43,44]. Empirical evidence of negative valence of both procedures in the majority of horses is very high, i.e., most horses avoid entering a trailer unless encouraged to, and horses have evolved to live in social groups and continue to avoid isolation from conspecifics [41]. The dimension of arousal is empirically less obvious, as some horses seem calmer than others and this may depend on many factors, including earlier life experiences and temperament [45].

Physical characteristics, such as age and sex, have been shown to have little effect on the physiological stress response in some instances [45] and even horses accustomed to transportation can show physiological changes characteristic of HPA activation [46]. However, sex and age could have a large impact on results when analyzing facial expressions. For example, the previous experience of the stressor, a factor strongly influenced by age, seems to decrease the response to some extent [45]. Irrespective of the horse’s previous experience, assessment of these interventions as emotionally stressful remains subjective. A rise in heart rate was observed during both the social isolation and transportation interventions in this study, which might indicate an increase in arousal/alertness or at least some form of physiological response, e.g. due to physical activity. It is also important to note that the increase is still within the limit of vagal variation within the horse, which makes conclusions strenuous. In the PRI group, earlier experience of transportation differed and some of the horses were even accustomed to travel by road on a weekly or monthly basis. Whether or not these horses experienced positive or negative valence to the transportation intervention is not known. This was one of the reasons for including the UNI subgroup, where all horses were unaccustomed to travel by road transport and all horses showed avoidance behaviors when being loaded. However, the results in this study should not be interpreted as a true measurement of the horses’ emotions, but rather as proof of changes in facial expressions due to high-arousal interventions. It is possible that other emotional states, such as excitement or fear, could be the source of the change in facial expressions and rise in heart rate observed during the interventions, meaning that the true emotional experience cannot be determined from the results in this study. The fact that horses react individually to transportation and social isolation is clearly illustrated in the results, with some horses showing little to no physiological changes during some interventions. The variance was generally higher in the PRI group, which probably reflects the heterogeneity of this population, both physically and mentally.

**Fig 4:**
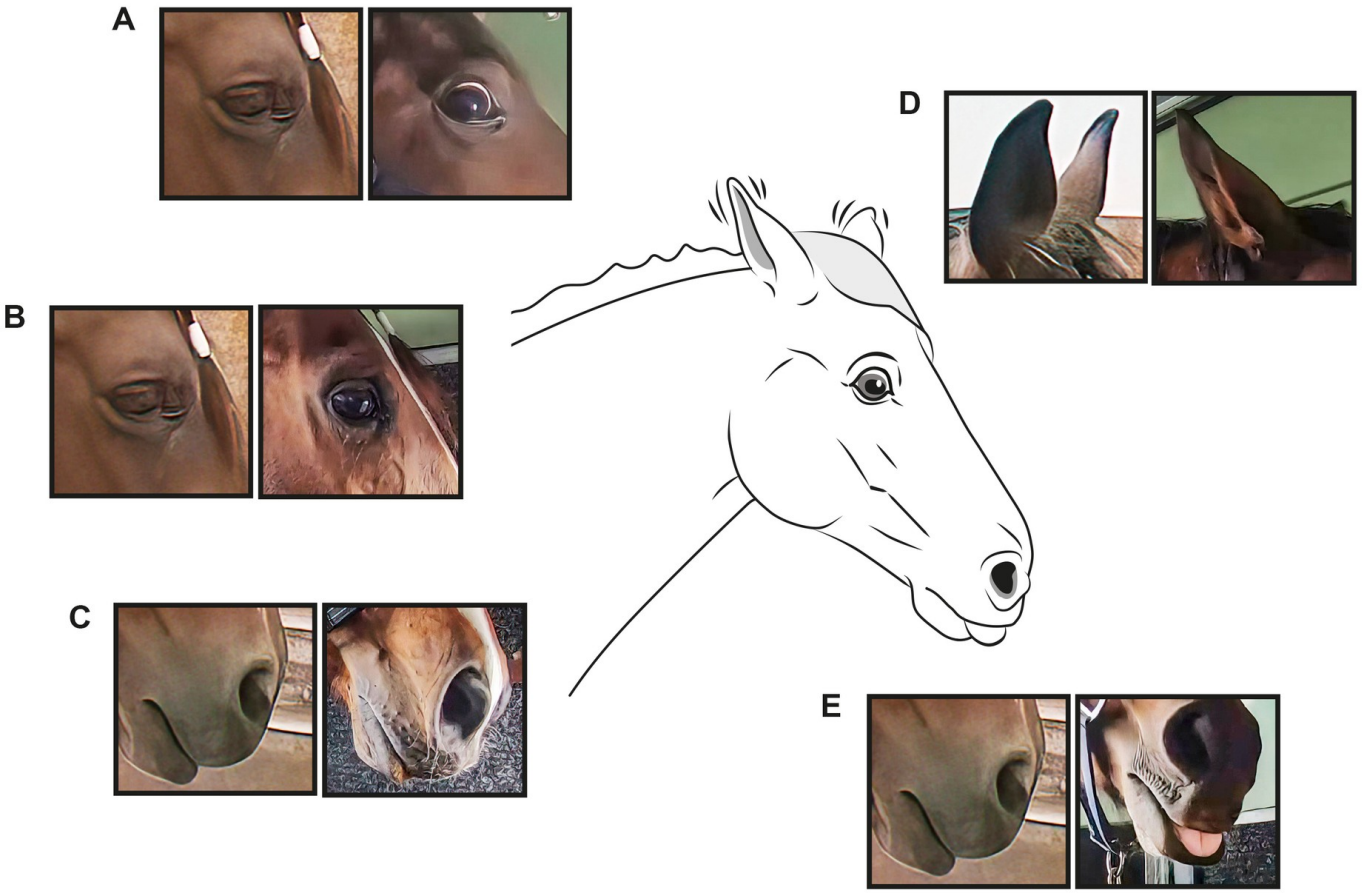
Illustration of facial expressions during the interventions. Action units (AU)/action descriptors (AD) relevant for the interventions. A: *Upper lid raiser* (AU5) and *Eye white increase* (AD1). B: *Inner brow raiser* (AU101). C: *Nostril dilator* (AD38). D: “Ear flicker”/ *Ear rotator* (EAD104). E: *Tongue show* (AD19). Action codes (right) are compared with a “neutral” horse (left). Illustration by Anders Rådén/ARDI.

Despite the large variation in our experimental horses, significant changes in facial activities were recorded after both transportation and social isolation (Fig 4). According to the HFI method, there was increased frequency of the AUs *upper lid raiser* (AU5) and *inner brow raiser* (AU101), as well as *blink* (AU145) and “ear flicker”. The frequency of the ADs *nostril dilator* (AD38) and *eye white increase* (AD1), not describing certain muscle-induced movements but rather the effects of two or more muscle movements, was also significantly increased. According to the Co-occurrence method, *tongue show* (AD19) and *mouth open* (AU25) were also important.

With this in mind, it is also possible that a number of external physical inputs inevitably associated with transportation, e.g., exposure to new environment, wind, confined space, ventilation or movement restriction, had some effect on the results presented in this study. In order to address this, the more homogeneous UNI subgroup underwent the social isolation intervention. Social isolation was associated with the same external inputs as the control, because the horses stayed in the same environment during the intervention. Changes in the facial expressions in this group should therefore be due to the experience of the horse being left alone, and not environmental factors. During social isolation, *Upper lid raiser* (AD5) and *Half blink* (AU47) were not selected. An explanation for this could be the environmental factors (wind, sounds, smells, movements) experienced during transportation. A distinct increase in the movement index “ear flicker” was apparent during both transportation and social isolation stress. Ear movements are very communicative [36], but during transportation ear movements due to sound might be a more likely cause of the high “ear flicker” frequency. During social isolation, a likely cause of ear movements is increased awareness of the surroundings due to arousal.

A reason for *upper lid raiser* (AU5) being more prominently seen during transportation stress, but not selected when analyzing isolation stress, could be that tension in *m*. *levator palpebrae superioris* (proposed basis for AU5) would hide tension in *m*. *levator anguli occuli medialis* (proposed basis for AU101) due to environmental factors but needs to be studied further. The frequency of *blink* (AU145) increased during both transportation and social isolation. An earlier study also reported an increase in blinks during stressful situations [47]. However, Merkies et al. [28] found that full blink diminished during stress. In the present study, the increase was only statistically significant for the Co-occurrence method during transportation stress. This may be a result of the greater number of horses in the transportation group. Differences in frequency of full blinks were not significant between baseline and the interventions (Fig 2). The only AU selected as indicative of a stressful intervention for the lower face was *lips part* (AU25). Concurrently, increased frequency of *tongue show* (AD19) was noted. This coincides well with earlier findings on behaviors of the tongue and repetitive mouth and licking behaviors during stress [34,48]. *Tongue show* (AD19) may be interpreted as a coping mechanism in horses subjected to stress, which is supported by the fact that oral stereotypies are often reported as a long-term consequence of inability to perform natural behavior in horses (e.g., cribbing).

When comparing the HFI method with the Co-occurrence method for two-second OWS, these two codes related to mouth movements were the only added codes. Since HFI is a frequency-based method, less frequent AUs such as *tongue show* (AD19) are not picked up using the HFI method, but were still sufficiently distinct to differentiate between stress and no-stress states. The logical interpretation of this pattern is that *tongue show* (AD19) and *lips part* (AU25) are sufficiently distinct to discriminate between stress and neutral states, but absence of the codes cannot exclude stress. This indicates the importance of the Co-occurrence method for selecting distinct and useful EquiFACS codes.

When comparing the facial expressions recorded in this study to facial activities previously described during stress in horses, similarities and differences were detected. Flared nostrils, repetitive mouth behaviors, increased eye white, and an increase in eye movements are features previously described during stressful interventions [23,24,30], However, increased activity of the *inner brow raiser* (AU101) is associated with pain in some pain assessment tools [22–24], and was not expected to be displayed during these management procedures where pain was not present. It is therefore relevant to discuss the possible presence of other states during the interventions. We recruited horses that were perceived as healthy and free from pain, and horses were used as their own control, so the risk of presence of pain in the majority of horses can be considered low, although not completely eliminated, since there is no ‘gold standard’ for evaluating pain.

The specificity of facial expressions across emotional states is of interest for their use as an emotional indicator [44]. To our knowledge, facial expressions during pain are the only experience to be analyzed to date using EquiFACS. Since pain is an internal stressor, while stress is not painful, comparison of facial expressions of pain and stress is needed. Rashid *et al*. [33] found that *nostril dilator* (AD38) and *chin raiser* (AU17) were indicative of pain when using both the HFI and Co-occurrence methods. The fact that *nostril dilator* (AD38) is also present during stressful management conditions could indicate that this AD is common during simple management interventions and less significant for determining pain. During both stress and pain, respiratory rate of the horse tends to increase, which may be a reason for *nostril dilator* (AD38) being common during both interventions.

As mentioned above, face-based pain scoring tools include facial expressions that were also present during the pain-free management interventions in this study. For example, the horse grimace scale [22] includes *ear flattener* (EAD103) and *ear rotator* (EAD104) as elements of the pain scale, while the FAP scale [24] uses eye white increase as an element. The “equine pain face” shows the features “tension of the lower face, rotated ears, dilated nostril and tension above the eye” [23]. All but “tension of the lower face” was seen in the pain-free stressed horses in this study. When discussing both physiological and behavioral aspects of pain assessment, stress is often described as a complicating factor [25]. This, together with our results, suggests a need for caution when using facial expressions for assessments during potentially stressful situations, since simple management procedures could induce similar facial expressions. Further, facial expressions during different emotional states should be studied in more controlled experiments in order to increase the validity of pain evaluations.

Despite great variation in the study group, the overall impressive recall and precision rates of the LOO classification indicate that the AUs/ADs selected by both the HFI method and the Co-occurrence method are indeed different from the baseline, and can successfully differentiate a high arousal state in horses from a control state. Interestingly, training the LOO classification on the videos with all AUs included generated almost as good results as only including relevant EquiFACS-codes selected by the HFI or Co-occurrence methods. This is probably because high-arousal states produced more facial activity, and therefore higher frequencies of EquiFACS codes, than the resting horse in its regular environment. However, on comparing two high-arousal states with many AUs, where specific features are more important to differentiate between states, the results would probably differ significantly more. Further studies should focus on using this method for comparing horses during high-arousal states during pain. Since these states both produce many AUs, this method could show promise in differentiating different affective states in the horse.

## Conclusions

It proved possible to induce and objectively record the presence of facial expressions in healthy horses under field conditions, using simple equipment and ordinary management practices. Applying two different frequency and duration-based methods revealed that two types of common management procedures (social isolation and transportation) induced increased frequencies of several facial movements. *Eye white increase* (AD1), *nostril dilator* (AD38), *inner brow raiser* (AU101), *upper lid raiser* (AU5), *tongue show* (AD19) and the facial movement index “ear flicker” were recorded when the horses underwent transportation and social isolation. These results partly corroborate earlier findings on behavioral aspects during stress. However, some of the facial activities (dilation of the nostril, contraction of m. *occulus levator angulii*) observed during pain-free transportation and social isolation are also commonly used in face-based pain assessment tools.

## Supporting information

S1 Dataset Data used for analysis. (XLSX)

S1 File HRM files (compressed R-R intervals for heart rate analysis). (ZIP)

S1 Video Sample clip of a baseline video. (MP4)

S2 Video Sample clip of an isolation video. (MP4)

S3 Video Sample clip of a transportation video. (MP4)
